# Highly Efficient Nanocarbon Coating Layer on the Nanostructured Copper Sulfide-Metal Organic Framework Derived Carbon for Advanced Sodium-Ion Battery Anode

**DOI:** 10.3390/ma12081324

**Published:** 2019-04-23

**Authors:** Chiwon Kang, Yongwoo Lee, Ilhwan Kim, Seungmin Hyun, Tae Hoon Lee, Soyeong Yun, Won-Sub Yoon, Youngkwang Moon, Jinkee Lee, Sunkook Kim, Hoo-Jeong Lee

**Affiliations:** 1 School of Advanced Materials Science and Engineering, Sungkyunkwan University (SKKU), Suwon 16419, Korea; seonkuk@skku.edu; 2 Department of Chemistry, University of Massachusetts Lowell, One University Avenue, Lowell, MA 01854, USA; Yongwoo_Lee@uml.edu; 3 Department of Applied Nano Mechanics, Korea Institute of Machinery and Materials (KIMM), Daejeon 305-343, Korea; kihwan20@kimm.re.kr (I.K.); hyun@kimm.re.kr (S.H.); 4 Center for Integrated Nanostructure Physics (CINAP), Institute for Basic Science (IBS), Suwon 16419, Korea; hooni0629@skku.edu; 5 Department of Energy Science, Sungkyunkwan University (SKKU), Suwon 16419, Korea; so116102@gmail.com (S.Y.); wsyoon@skku.edu (W.-S.Y.); 6 School of Mechanical Engineering, Sungkyunkwan University, Suwon 16419, Korea; fire6568@skku.edu (Y.M.); lee.jinkee@skku.edu (J.L.)

**Keywords:** copper sulfide (Cu_x_S), metal organic framework (MOF), sodium ion battery (SIB), carbon coating layer, nanoporous anode materials, high specific surface area, nanostructured anode, H_2_S gas-assisted plasma-enhanced chemical vapor deposition (PECVD), sulfurization, carbonization

## Abstract

High theoretical capacity and low-cost copper sulfide (Cu_x_S)-based anodes have gained great attention for advanced sodium-ion batteries (SIBs). However, their practical application may be hindered due to their unstable cycling performance and problems with the dissolution of sodium sulfides (Na_x_S) into electrolyte. Here, we employed metal organic framework (MOF-199) as a sacrificial template to fabricate nanoporous Cu_x_S with a large surface area embedded in the MOF-derived carbon network (Cu_x_S-C) through a two-step process of sulfurization and carbonization via H_2_S gas-assisted plasma-enhanced chemical vapor deposition (PECVD) processing. Subsequently, we uniformly coated a nanocarbon layer on the Cu_1.8_S-C through hydrothermal and subsequent annealing processes. The physico-chemical properties of the nanocarbon layer were revealed by the analytical techniques of high-resolution transmission electron microscopy (HRTEM), energy-dispersive X-ray spectroscopy (EDS), and scanning electron microscopy (SEM). We acquired a higher SIB performance (capacity retention (~93%) with a specific capacity of 372 mAh/g over 110 cycles) of the nanoporous Cu_1.8_S-C/C core/shell anode materials than that of pure Cu_1.8_S-C. This encouraging SIB performance is attributed to the key roles of a nanocarbon layer coated on the Cu_1.8_S-C to accommodate the volume variation of the Cu_1.8_S-C anode structure during cycling, enhance electrical conductivity and prevent the dissolution of Na_x_S into the electrolyte. With these physico-chemical and electrochemical properties, we ensure that the Cu_1.8_S-C/C structure will be a promising anode material for large-scale and advanced SIBs.

## 1. Introduction

Sodium ion batteries (SIBs) have drawn great attention as an alternative to lithium ion batteries (LIBs) for large-scale energy storage systems (ESS) such as home solar-power storage, microgrids, and load leveling, owing to the abundant resource and low cost of Na [1,2]. However, the Na^+^ ion has a larger ionic radius of 1.06 Å and a heavier atomic weight of 23 g/mol relative to the 0.76 Å and 6.9 g/mol of the Li^+^ ion [3]. These immutable properties have raised critical issues on sluggish Na^+^ ion kinetics caused by Na^+^’s large migration energy barrier, thereby leading to the unsatisfactory electrochemical performance of electrode materials (e.g., graphite-based electrode) with the intercalation/deintercalation mechanism of the Na^+^ ion. 

As an alternative electrode material to overcome these bottlenecks, transition metal oxides (TMOs) [4] and sulfides (TMSs) [5] have been vigorously investigated as intercalation/conversion-type anode materials owing to their high theoretical capacity (400–560 mAh/g) [6]. Compared to TMO-based electrodes, TMS-based electrodes possess their own beneficial properties including their relatively small volume change, superior reversibility, and higher electrical conductivity because of proportionally-weaker M–S ionic bonds relative to their M–O counterparts, which enables enhanced rechargeable battery performance [7]. Among the TMS-based anode materials, cuprous sulfide (Cu_x_S) has been intensively investigated as a strong candidate electrode material for LIBs [8,9,10,11,12,13,14,15,16,17] and SIBs [3,7,18,19], owing to its unique properties of high theoretical capacity (~560 mAh/g for CuS and 337 mAh/g for Cu_2_S), low cost, the environmental harmlessness of sulfur, and good electrical conductivity (10^−3^ S/cm) [7,20]. Although the Cu_x_S-based electrodes have been extensively studied, they have posed some major issues including [3,7,18,19]: (1) irreversible conversion reaction between Cu_x_S and the Li^+^/Na^+^ ion, which induces a large volume variation of the Cu_x_S-based electrodes during prolonged cycling, bringing about corruption of structural integrity and capacity decay; (2) dissolution of sodium sulfide (Na_x_S) into electrolytes during cycling, causing the loss of active materials. The most viable approaches to overcome these limitations include nanostructure design and subsequent incorporation of nanocarbon (e.g., carbon nanotubes [21], reduced graphene oxide [9,22], and mesoporous carbon [8]) with Cu_x_S, electrolyte selection [23], and voltage range modification [3]. Metal organic frameworks (MOF) are a subclass of nanoporous coordination polymers composed of molecular building blocks comprising metal ions that are linked by organic linker ligands [24]. Because of MOF’s appealing properties of crystallinity, along with its high surface area and porosity, these materials have been widely implemented into gas sorption/separation [25], toxic gas mitigation [26], catalysis [27], batteries, and supercapacitors [28,29]. Very recently, highly nanoporous MOFs were employed as a sacrificial template to synthesize Cu_x_S-carbon nanocomposite cathode materials through thermal evaporation of sulfur powders for LIBs [14,30]. Nevertheless, their cycling stability still needs to be improved [12] along with their specific capacity [28]. What remains largely unexplored to overcome these limitation is the design and fabrication of a Cu_x_S-MOF-derived carbon/nanocarbon (Cu_x_S-C/C) core/shell anode structure. In this structure, nanocarbon jointly surrounding the Cu_x_S-C could buffer against mechanical stresses derived from a large volume change of Cu_x_S during the sodiation-desodiation cycling process and mitigate a problem associated with structural pulverization of the Cu_x_S, thus enhancing the cycling performance. Furthermore, the nanocarbon is able to improve the electrical conductivity of the Cu_x_S and prevent the dissolution of Na_x_S into electrolytes during cycling.

Inspired by this, we embarked on designing and fabricating the nanoporous Cu_x_S-C network with a high surface area to incorporate a carbon nanolayer by producing a core-shell nanostructure through a two-step process. First, we synchronized sulfurization and carbonization via a H_2_S gas-assisted plasma-enhanced chemical vapor deposition (PECVD) process, in which it is possible for Cu^2+^ ions in the core of a single MOF particle to be sulfurized at room temperature, thus forming CuS [25,26]. Furthermore, this process is more efficient when compared to solution processing for CuS microsphere synthesis [7]. Second, we coated the nanocarbon layer on the surface of the Cu_x_S-C. Furthermore, we applied the cut-off voltage range (0.01–3 V) for the battery test without compromising specific capacity, which differs from the previous report [3]. Digenite Cu_1.8_S-C (showing the highest capacity among the polymorphs of Cu_x_S investigated in this study) was coated by a nanocarbon layer to form a core/shell structure, demonstrating a specific capacity of 372 mAh/g at 2C over 110 cycles, superior to that of the pure Cu_1.8_S-C anode, and a higher cycling stability with ~93% retention. Considering these results, we ensure that the highly nanoporous Cu_1.8_S-C/C core/shell anode structure can shed light on resolving the remaining issues of Cu_x_S-based anodes for large-scale, next-generation SIBs.

## 2. Materials and Methods

### 2.1. Synthesis of MOF-199

We fabricated metal organic framework (MOF-199) with an octahedral shape through a simple solvothermal synthesis method. In the typical synthesis, Cu_3_(NO_3_)_2_·3H_2_O (Sigma-Aldrich, St. Louis, MI, USA) as a Cu-precursor (334.83 mg; 1.785 mmol), 1,3,5-benzenetricarboxylic acid (trimesic acid, H3BTC, C_6_H_3_(CO_2_H)_3_) (Sigma-Aldrich) as an organic linker (196 mg; 0.933 mmol), and lauric acid (Fisher Scientific, Hampton, NH, USA) as a growth modulator of the MOF-199 structure (9.917 g; 49.5 mmol) were dissolved into n-butyl alcohol (70 mL) and then sonicated for 1 h to acquire a homogeneous solution using a sonicator with 40-kHz sound waves (Lab Companion; UC-10, JEIO TECH, Daejeon, Korea). The solution was subsequently transported into a 100-mL Teflon-lined stainless steel autoclave, which was pressurized at 140 °C for 5 h. After synthesis, we purified the as-synthesized MOF-199 particles via centrifugation with ethanol and deionized (DI) water several times. Finally, the purified MOF-199 particles were dried at 80 °C overnight in a drying oven. Figure S1 illustrates a dicopper (II) tetracarboxylate building block for MOF-199 [31].

### 2.2. Synthesis of Cu_x_S-MOF-Derived Carbon

Five hundred milligrams of MOF-199 were subjected to a two-step process of sulfurization and carbonization. H_2_S (10%)/N_2_ (90%) mixture gas (30 sccm) and Ar gas (100 sccm) were flowed into a quartz tube in the PECVD system. Plasma power (100 W) was applied to decompose H_2_S gas into atomic S and H, ions of S^2−^, S^−^, and H^+^, radical of H_2_S^+^, and molecular H_2_ at the temperature ranging from 350–650 °C at a rate of 5 °C/min [32]. Among these species, the S^2−^ radical was reacted with Cu^2+^ ions coordinated with the organic linker, forming nanostructured CuS even at room temperature [25,26]. The process pressure was retained as ~1.0 torr. During the sulfurization process, the original MOF-structure was dismantled through thermal decomposition and sulfurization of other elements (e.g., oxygen and hydrogen) in the original MOF unit structure, finally forming the nanocarbon network [14,33]. Further sulfurization and carbonization proceeded at the respective temperature (350, 550, and 650 °C) for 1 h. Finally, rapid cooling was carried out in an Ar gas environment at 100 standard cubic centimeter per minute (sccm).

### 2.3. Synthesis of Cu_1.8_S-MOF-Derived Carbon/Carbon Core/Shell Structure

**Figure 1:**
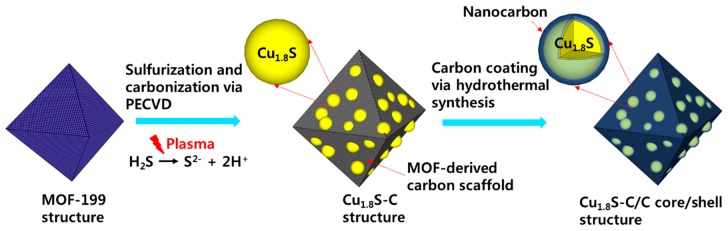
The schematic diagram representing each synthesis process for the Cu_1.8_S-C/C core/shell structure.

Cu_1.8_S (77 wt%)-MOF-derived carbon (23 wt%) sulfurized at 550 °C (200 mg; 1.1 mmol) and dextrose (Sigma-Aldrich) (400 mg; 2.2 mmol) were dissolved into 12.5 mL of de-ionized (DI) water. Following this, the solution was sonicated for 30 min for homogeneous mixing. Subsequently, the solution was put into a 25-mL Teflon-lined autoclave for hydrothermal synthesis at 190 °C for 12 h. After completing the hydrothermal synthesis, the resulting powder sample was purified through centrifugation with DI water several times. Finally, the purified powder sample was subjected to the annealing process at 500 °C for 8 h under a Ar/H_2_ (90/10 wt%) mixture gas environment. Figure 1 schematically illustrates each synthesis procedure for the Cu_1.8_S-C/C core/shell structure.

### 2.4. Structural Analysis of Cu_x_S-C/C

The phase identification of the MOF-199 and Cu_x_S-C was acquired by X-ray diffraction (XRD) (Bruker, Billerica, MA, USA; D8 ADVANCE). The morphology and structural features were observed using scanning electron microscopy (SEM) (JEOL, Akishima, Japan; JSM-7600F) and Cs corrected S/transmission electron microscopy (TEM) (JEOL, Akishima, Japan; JEM-ARM200F). The quality of nanocarbon in the Cu_1.8_S-C/C core/shell structure was measured by Raman spectroscopy (NT-MDT, Moscow, Russia; Ntegra Spectra DUO Max). Additionally, the thermal behavior of the MOF-199 and Cu_x_S-C samples and the weight contents of the Cu_x_S in each Cu_x_S-C sample were measured by using thermogravimetry (TG)-differential thermal analysis (DTA) (SEICO INST, Chiba, Japan; TG/DTA7300). The specific surface area and pore volume of the MOF-199 and Cu_1.8_S-C/C were measured by using Brunauer-Emmett-Teller (BET) equipment (Micromeritics, Norcross, GA, USA; ASAP2020). Micropore and meso-/macro-pore size distributions were evaluated through the Horvath–Kawazoe (HK) and Barrett-Joyner-Halenda (BJH) methods, respectively. The chemical composition, electronic states and purity of the Cu_1.8_S-C/C sample were analyzed by using X-ray photoelectron spectroscopy (XPS) (Thermo Fisher Scientific, Waltham, MA, USA; K-alpha).

### 2.5. Sodium Ion Battery Performance of Cu_x_S-C and Cu_1.8_S-C/C Anode Structures

The electrode slurry was fabricated by mixing active materials (80 wt%), graphene nanoplatelets with a surface area of 500 m^2^/g (Sigma-Aldrich) (10 wt%) as a conducting agent, and polyvinylidene difluoride (PVDF) (Sigma-Aldrich) (10 wt%) as a binder in the solvent of *n*-methyl-2-pyrrolidone (Sigma-Aldrich). The slurry was coated onto a copper current collector and then dried at 80 °C in a drying oven for 12 h. The coin-type of cells composed of the Cu_x_S-C and Cu_1.8_S-C/C as the working electrodes were assembled with cell components (Welcos, Seoul, Korea) in a glove box under an inert Ar gas environment. Coin-cells were assembled with a porous glass fiber (grade GF/F, Whatman, Little Chalfont, U.K.) film as a separator, 1 M sodium trifluoromethanesulfonate (NaSO_3_CF_3_) (Sigma-Aldrich) in diethylene glycol dimethyl ether (DEGDME) (Sigma-Aldrich) as an electrolyte, and a sodium (Sigma-Aldrich) foil electrode as counter and reference electrodes. The galvanostatic charge and discharge cycling test was carried out by using a multichannel battery testing unit (CTS-Lab, Asselfingen, Germany; BaSyTec). The cyclic voltammetry (CV) for the Cu_1.8_S-C/C anode sample was conducted using a multi-channel potentiostat (Bio Logic, Seyssinet-Pariset, France; VMP3) in the voltage range of 0.01–3.0 V (vs. Na^+^/Na) at a scan rate of 0.2 mV/s.

## 3. Results and Discussion

### 3.1. Structural and Morphological Properties of MOF-199, Cu_x_S-C and Cu_1.8_S-C/C Core/Shell Structures

**Figure 2:**
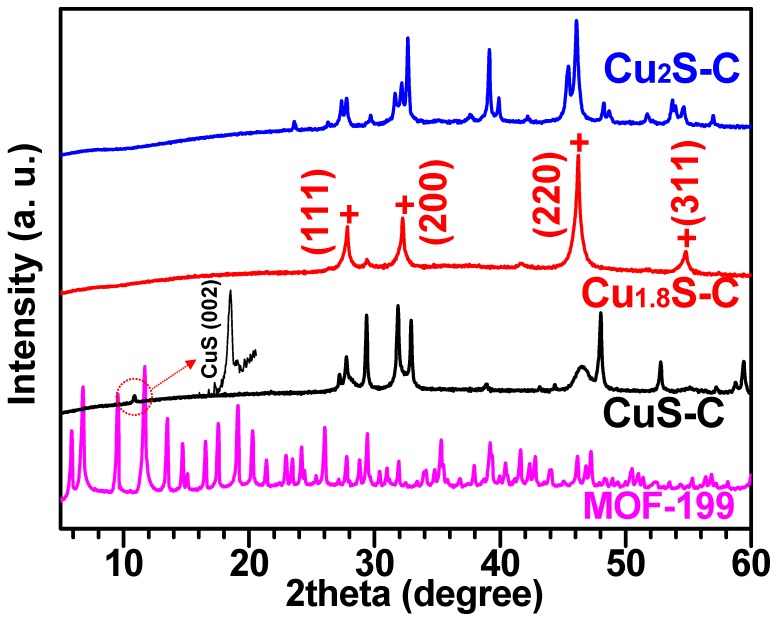
XRD patterns of the MOF-199 and three different samples of Cu_x_S. Note that intensity is in logarithmic scale. We assign the (002) reflection at ~10° in the CuS-C sample as “CuS (002)”.

Figure 2 shows the XRD patterns of the MOF-199 and three different Cu_x_S samples. The peaks of XRD for the samples subjected to the different sulfurization conditions were consistent with the hexagonal covellite CuS (PDF#06-0464) at 350 °C, digenite Cu_1.8_S (PDF#47-1748) at 550 °C and chalcocite Cu_2_S (PDF#84-1770) at 650 °C, respectively. There was no other peak regarding MOF-199, sulfur, Cu_2_O, and impurities after the two-step process of sulfurization and carbonization, highlighting that the as-synthesized MOF-199 was completely transformed into the copper sulfide (Cu_x_S) that was incorporated in the MOF-derived carbon network. Noticeably, the vaporization degree of sulfur proportionally increased with sulfurization temperature from 350–650 °C, thus transforming the copper-poor (CuS) to the copper-rich (Cu_1.8_S and Cu_2_S) phases with temperature [20]. For the cubic structure of Cu_1.8_S, we could identify refined lattice constants (a = b = c = 5.55 Å) according to Bragg’s law, similar to the reported value of Cu_1.8_S [3]. We observed no peak corresponding to the carbon in the Cu_x_S-C samples, which is probably due to the screening effect induced by the Cu_x_S. Alternatively, we proved the presence of the carbon by using EDS elemental mapping and spectra data (Figures S2–S4).

**Figure 3:**
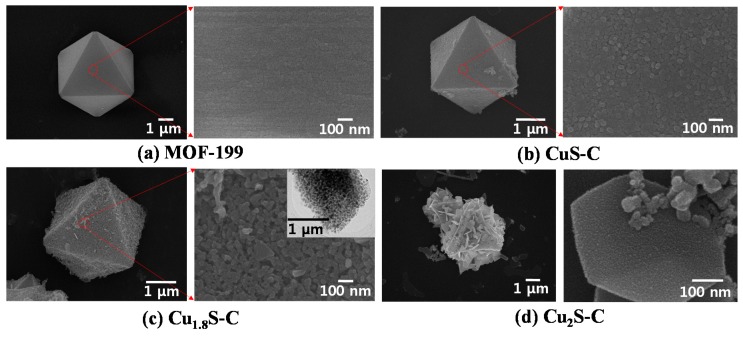
SEM images showing the morphological characteristics of as-synthesized MOF-199 and the degree increment of Cu_x_S samples and their corresponding morphological variation with sulfurization and carbonization temperature through the H_2_S gas-assisted PECVD process. (**a**) MOF-199; (**b**) CuS-C at 350 °C; (**c**) Cu_1.8_S-C at 550 °C; (**d**) Cu_2_S-C at 650 °C.

Figure 3a demonstrates the SEM images showing the morphologies of as-synthesized MOF-199 with an octahedral shape used as a sacrificial template for Cu_x_S embedded in a conductive nanocarbon scaffold. EDS mapping and spectra results of the as-synthesized MOF-199 structure proved that the constituent elements (e.g., Cu, O, and C) were uniformly distributed in the entire region of the structure (see Figure S5). SEM images presented in Figure 3b–d demonstrate the morphological variations of the Cu_x_S with the sulfurization temperature from 350–650 °C. We can observe a larger number of Cu_x_S nanoplates protruding from the surface at higher temperature, thus confirming that the degree of Cu_x_S growth was proportionally increased with the sulfurization temperature. The high magnification SEM image of Figure 3c exemplifies the uniformly-distributed interconnected/aggregated Cu_1.8_S nanoparticles in the carbon network. The inset TEM image in Figure 3c identifies the average size (~60 nm) of Cu_1.8_S nanoparticles, which is consistent with the nanoparticle distribution presented in Figure 3c. At 650 °C, the intensive sulfurization process increased the growth rate of Cu_2_S nanoflakes, hence causing the collapse of an overall octahedrally-shaped structure as exhibited in the higher magnification SEM image in Figure 3d. 

### 3.2. Thermogravimetric Analysis of MOF-199 and Cu_x_S-C Structures

**Figure 4:**
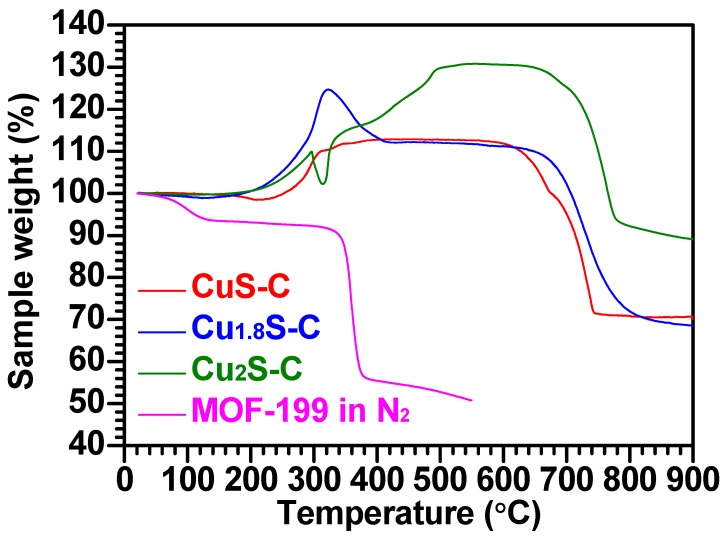
TG curves of CuS-C, Cu_1.8_S-C, and Cu_2_S-C in air and MOF-199 in a N_2_ gas environment.

Figure 4 demonstrates the thermal behavior of the MOF-199 and Cu_x_S-C in the temperature ranging from 20–900 °C. The MOF-199 was thermally degraded under an inert nitrogen (N_2_) gas environment, hence resulting in a weight decrease (~36%) at around 350 °C according to the TG curve. This weight loss is attributed to the decomposition of organic linker (C_6_H_3_(CO_2_H)_3_), which occurred around 300 °C. The three Cu_x_S-C samples were subjected to the TG analysis in an air atmosphere to identify the contents of Cu_x_S in each Cu_x_S-C sample. Figure 4 shows the three different Cu_x_S polymorphs undergoing the multi-step reactions with oxygen in air in the temperature ranging from 20–900 °C. The detailed reaction mechanism is presented in the Supplementary Information [14]. Based on the mechanism, we can calculate the real contents of CuS, Cu_1.8_S, and Cu_2_S in each Cu_x_S-C structure as ~46, ~77, and ~91 wt%, respectively.

### 3.3. Structural and Morphological Properties of Cu_1.8_S-C/C Core/Shell Structures

**Figure 5:**
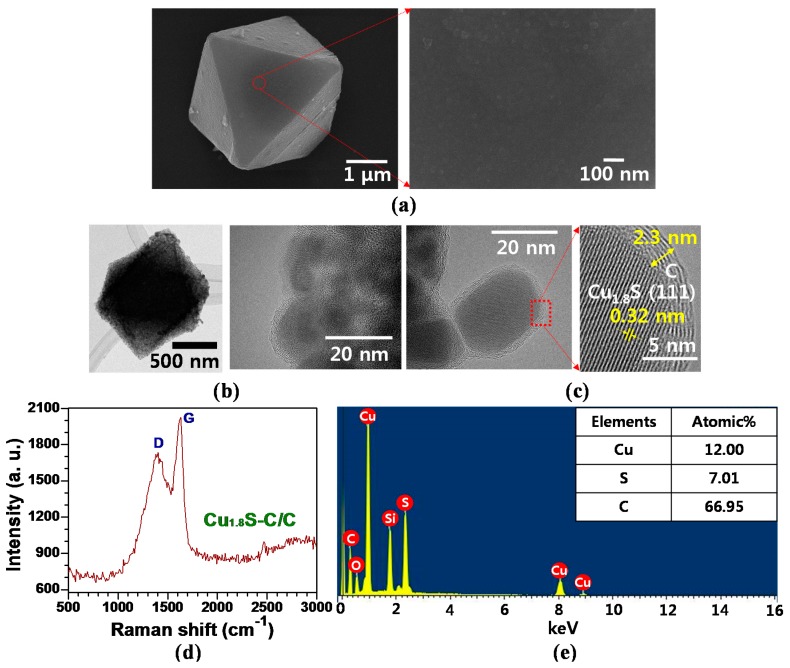
(**a**) High and low magnification SEM images showing the morphological features of the Cu_1.8_S-C/C core/shell structure; (**b**) TEM images of an octahedral Cu_1.8_S-C/C sample; (**c**) high resolution TEM (HR-TEM) pictures to reveal the core/shell structure of the Cu_1.8_S-C/C sample, along with the interlayer distance of the Cu_1.8_S (111) plane and the zoom-in image of the glucose-based carbon coating layer, denoted as C; (**d**) Raman and (**e**) EDS spectra of the Cu_1.8_S-C/C sample.

The MOF-derived carbon networks play an important role in enhancing the electrical conductivity of Cu_x_S-C samples and buffering against structural variation during charge and discharge cycles. Figure 5a reveals the characteristic surface of Cu_1.8_S-C with the glucose-based carbon coating layer. Compared to the Cu_1.8_S-C without carbon coating (Figure 3c), the individual morphologies of Cu_1.8_S nanoparticles were less distinguishable since the nanocarbon layer was coated on the surface, and then, the nanoporous regions among them were effectively filled with the coated carbon. The TEM images in Figure 5b highlight a single Cu_1.8_S-C/C core-shell structure (lower magnification image) and Cu_1.8_S nanoparticles embedded in the carbon network (higher magnification image). We can distinguish between Cu_1.8_S nanoparticles and low-degree graphitic carbon by the contrast difference (the brighter region corresponds to the carbon, whereas the darker region the Cu_1.8_S), which is consistent with the TEM image shown in the inset of Figure 3c. Figure 5c more clearly shows the Cu_1.8_S-C/C core-shell structure. The high resolution TEM image helps us to measure the interlayer distance (~0.32 nm) of the Cu_1.8_S (111) plane in the core-region and the thickness (~2.3 nm) of the carbon coating layer in the shell-region. Figure S6 displays an HRTEM image showing the interlayer distance (~0.27 nm) of the Cu_1.8_S (200) plane and the thickness (~2.6 nm) of the carbon layer. Figure S7 illustrates the EDS line scanning results of a Cu_1.8_S-C/C core-shell structure to identify the distribution of S and C elements comprising the structure. Note that the results of elemental Cu have low reliability due to the noise-inducing effects of the Cu grid on the detection of accurate results. Raman spectra results exhibited the presence of the carbon layer coated on the Cu_1.8_S-C/C core-shell structure according to the typical G– and D–band peaks appearing at ~1580 and ~1350 cm^−1^ (Figure 5d). Their peak ratio (I_D_/I_G_) reached to ~0.85, which indicates low-degree graphitic carbon [34]. The visible inner carbon structure is attributed to the organic linker decomposition, whereas the external carbon structure to the conversion from glucose to the amorphous carbon through the hydrothermal synthesis and subsequent annealing process. For comparison purposes, we calculated the I_D_/I_G_ peak ratio (~0.93) of the MOF-derived inner carbon of the Cu_1.8_S-C sample (Figure S8); this value is comparable to that of the Cu_1.8_S-C/C sample comprising both glucose-derived external and MOF-derived inner carbons. These results suggest that both external and inner carbons belong to the low-degree graphitic carbon without significant structural distinctions between them. The EDS spectra in Figure 5e exhibit the presence of each element (Cu, S, and C) comprising the Cu_1.8_S-C/C core/shell particle sample. Note that Si and O peaks are related to the Si/SiO_x_ substrate used for SEM image acquisition of the particles. The atomic ratio of Cu to S is measured as ~1.7, which is close to the ratio of Cu_1.8_S. 

### 3.4. Surface Area and Porosity of MOF-199 and Cu_1.8_S-C/C Core/Shell Structures

**Figure 6:**
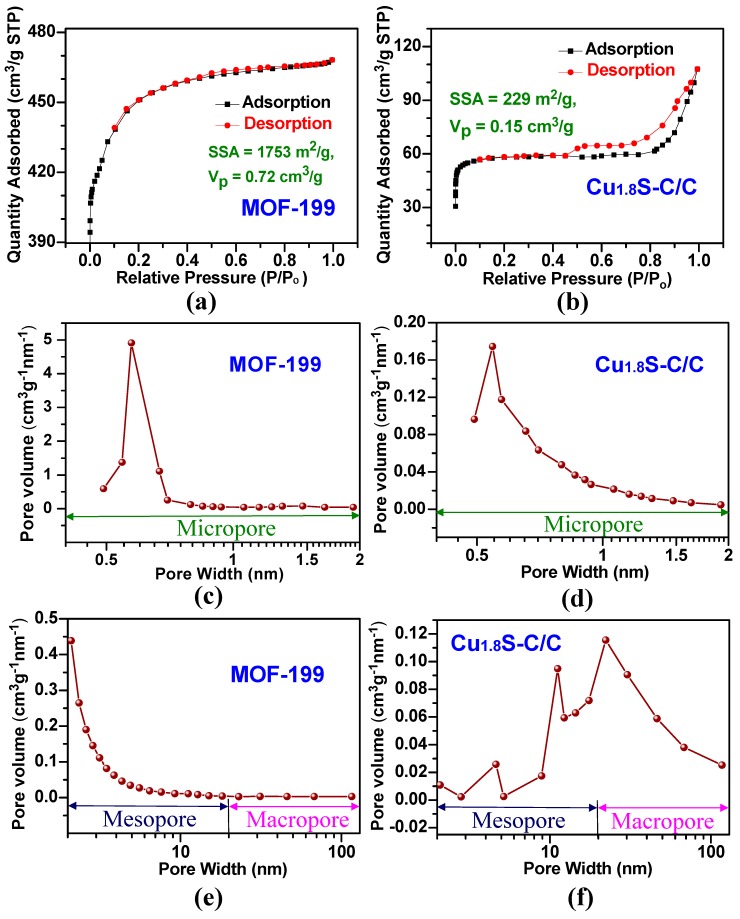
(**a**,**b**) Nitrogen adsorption–desorption isotherms of the MOF-199 and Cu_1.8_S-C/C structures; (**c**–**f**) pore size distribution curves of the MOF-199 and Cu_1.8_S-C/C structures. Note that pore width is in logarithmic scale.

The large surface area and porosity of Cu_1.8_S-C/C could promote sodium ion diffusion in the structure and increase the available sites for sodium ion accommodation during the charge and discharge cycle, thereby enhancing SIB performance [30]. To prove the correlation between high porosity and the large surface area of the Cu_1.8_S-C/C and its electrochemical performance, we analyzed the N_2_ adsorption and desorption isotherms and pore-size distribution curves of the MOF-199 and Cu_1.8_S-C/C, respectively (see Figure 6). The MOF-199 sample illustrated a typical type-I isotherm defined by IUPAC (International Union of Pure and Applied Chemistry) as the steep rise of the uptake occurred at low N_2_ relative pressure. This is because of the filling of N_2_ in the micropores followed by a plateau region, which is representative of a microporous material (Figure 6a) [35]. Conversely, the Cu_1.8_S-C/C sample exhibited an isotherm with a type-H3 hysteresis loop, according to the IUPAC definition, due to the low volume (~59 cm^3^/g at standard temperature and pressure (STP)) adsorbed at low relative pressure (P/P_0_ < 0.45). This result could be attributed to the low specific surface area (229 m^2^/g) and pore volume (0.15 cm^3^/g). Furthermore, the isotherm of the Cu_1.8_S-C/C sample showed an additional adsorption volume of N_2_ gas at the higher relative pressure level (P/P_0_ > 0.5) (Figure 6b) as the Cu_1.8_S-C/C sample had broad distributions of slit-shaped meso-/macro-pores with a pore volume of 0.08 cm^3^/g, thus supporting the type-H3 hysteresis loop (Figure 6f) [36]. The MOF-199 exhibited a sharp peak with a maximum at 0.57 nm showing a micropore volume (0.61 cm^3^/g) in the micropore range (Figure 6c), whereas the Cu_1.8_S-C/C a (slightly shifted) maximum peak at 0.54 nm (Figure 6d), suggesting that the micropores (pore volume: 0.07 cm^3^/g) of the Cu_1.8_S-C/C were retained after the sulfurization and carbonization process. Furthermore, the MOF-199 showed a broad peak in the range of 2–10 nm, indicating the presence of mesopores (pore volume: 0.11 cm^3^/g) in the structure (Figure 6e). Furthermore, the specific surface areas of the MOF-199 and Cu_1.8_S-C/C samples were measured as 1753 (1517 (micropore) + 236 (meso-/macropore)) and 229 (206 (micropore) + 23 (meso-/macropore)) m^2^/g, respectively. The decrease in the pore volume and specific surface area of the Cu_1.8_S-C/C stems from the microstructural variation occurring during the sulfurization and carbonization process. Despite this, the Cu_1.8_S-C/C somewhat retained the MOF-199-derived micropores and the newly-generated mesopores. It should be noted that the Cu_1.8_S-C/C had a higher pore volume and specific surface area compared to the previous reports of Cu_x_S-C/C [20,30,33]. These results suggest that the Cu_1.8_S-C/C could offer facilitated Na^+^ ion diffusion and reaction, inter-space volume to accommodate volume change, and a large contact area of the electrode/electrolyte interface during cycling process, thus leading to enhanced SIB performance [18].

### 3.5. X-ray Photoelectron Spectroscopy Analysis of Cu_1.8_S-C/C Core/Shell Structure

**Figure 7:**
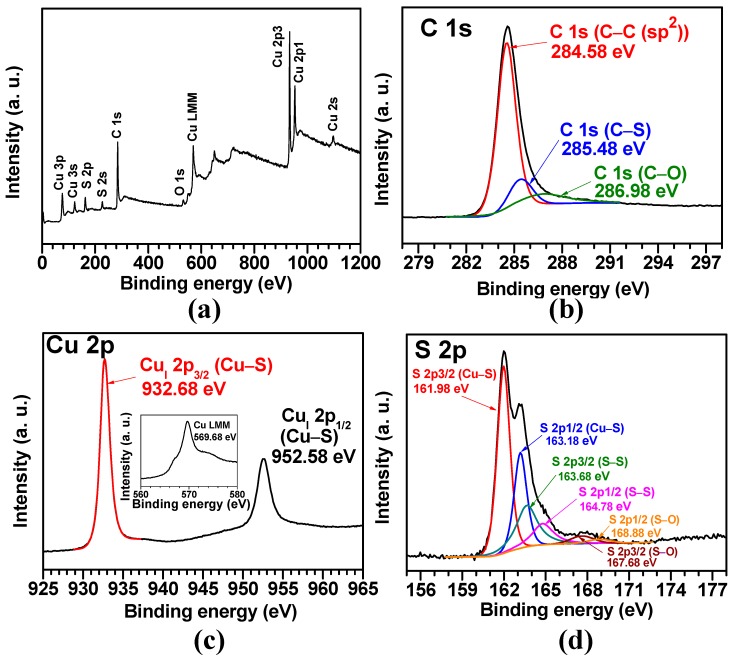
XPS spectra of the Cu_1. 8_S-C/C: (**a**) survey spectra; high-resolution spectra of (**b**) C 1s, (**c**) Cu 2p, and (**d**) S 2p.

To study the chemical composition, electronic state, and purity of the Cu_1.8_S-C/C sample, we conducted XPS analysis. Figure 7a demonstrates a wide-scan XPS spectra, proving the presence of Cu, S, C, and O elements with their characteristic binding energies calibrated by referencing C 1s sp^2^ bonding (284.58 eV shown in Figure 7b). Figure 7c exhibits the two strong peaks at 932.68 and 952.58 eV, associated with Cu_(I)_ 2p_3/2_ and Cu_(I)_ 2p_1/2_, respectively, and there exists no satellite peak related to Cu^2+^, thus proving only Cu^+^ exists in the Cu_1.8_S [37]. The inset of Figure 7c presents the Cu LMM Auger transition with a maximum peak at 569.68 eV, which is the typical binding energy value for Cu_1.8_S [38]. Figure 7d shows S 2p spectra, completed by data fitting, with a characteristic S 2p doublet for S^2−^ with S 2p_3/2_ and S 2p_1/2_ peaks appearing at 161.98 and 163.18 eV, respectively [39]. The binding energy (161.98 eV) of S 2p_3/2_ could correspond to S atoms bonded to Cu (S–Cu), forming Cu_1.8_S. The shoulder peaks appearing at 163.68 and 164.78 eV are related to S atoms in S–S bonds [38]. The binding energy peaks at 167.68 and 168.88 eV can be due to SO_4_^2−^ groups [40] formed by surface oxidation during the sulfurization process. No other impurity peak was observed, suggesting the Cu_1.8_S-C/C sample was relatively pure. 

### 3.6. Electrochemical Performance of Cu_x_S-C and Cu_1.8_S-C/C Core/Shell Structures

**Figure 8:**
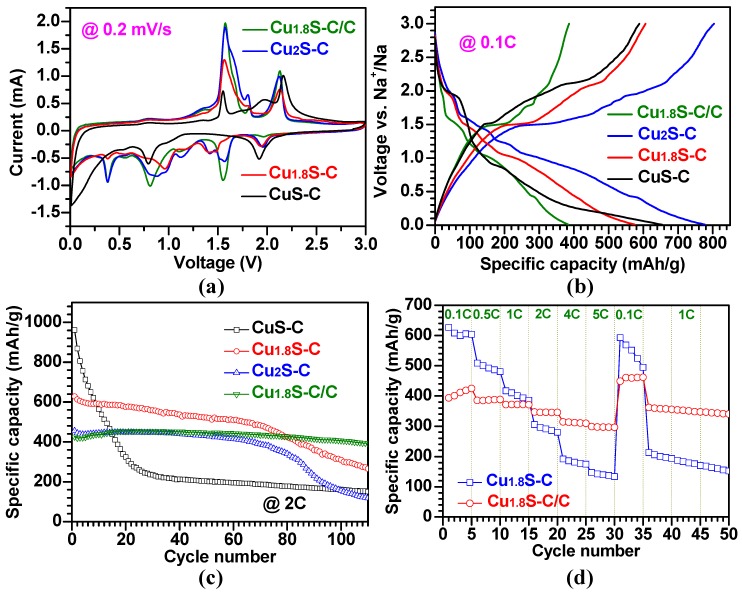
Electrochemical performance of the Cu_x_S-C and Cu_1.8_S-C/C anode structures. (**a**) CV curves of the Cu_x_S-C and Cu_1.8_S-C/C anode structures in the voltage range of 0.01–3 V at a scan rate of 0.2 mV/s for the second cycle; (**b**) characteristic voltage profiles of the Cu_x_S-C and Cu_1.8_S-C/C anode structures; (**c**) cycling performance of the Cu_x_S-C and Cu_1.8_S-C/C anode structures; (**d**) comparative study on C-rate capability between the Cu_1.8_S-C and Cu_1.8_S-C/C anode structures.

Figure 8 demonstrates the SIB performance of Cu_x_S-C and Cu_1.8_S-C/C structures with the aforementioned physico-chemical properties. Figure 8a shows the cyclic voltammetry (CV) curves for the Cu_x_S-C and Cu_1.8_S-C/C anode samples for the second charge-discharge cycle. For all the samples, the two main oxidation (anodic) peaks occurred at ~1.6, and ~2.1 V, while the three main reduction (cathodic) peaks appeared at ~0.8, ~1.5, and ~1.9 V. These redox peaks, in conjunction with the other peaks, are associated with the multi-step electrochemical reactions for sodiation/desodiation process. Furthermore, these peaks correspond to the plateaus shown in the voltage profile curves of the same sample (Figure 8b), consistent with the previous reports of Cu_x_S-based anodes [3,7,18,19]. In Figure 8c, cycling performance results of the Cu_1.8_S-C/C sample demonstrate that the specific capacity increased from 402 to 426 mAh/g for the initial 20 cycles due to its microstructural changes [16], which can offer additional sites for Na^+^ ion accommodation and higher ionic diffusivity, and then decreased to 372 mAh/g (~93% capacity retention) over 110 cycles. This value was superior to those of previous reports [3,7,18,19] and ~140% higher than the pure Cu_1.8_S-C. The capacity reversibility (Coulombic efficiency) was measured as nearly 100% (see Figure S9). The specific capacity of the Cu_1.8_S-C sample was higher than the Cu_1.8_S-C/C up to 78 cycles; however, it kept decreasing from 628 to 266 mAh/g (~42% capacity retention) over the entire cycle process. Furthermore, the CuS-C and Cu_2_S-C samples showed specific capacities of 155 and 110 mAh/g after 110 cycles and their corresponding capacity retentions of ~16 and ~26%, respectively. The superior capacity retention with higher specific capacity of the Cu_1.8_S-C/C is attributed to the role of the nanocarbon layer covering the Cu_1.8_S-C structure. This layer is thought to buffer against the mechanical stress caused by volume variation of the Cu_1.8_S structure during cycling and to alleviate a structural pulverization of the Cu_1.8_S, therefore enabling the improved cycling performance. Furthermore, the nanocarbon can enhance the electrical conductivity of the Cu_1.8_S and tether sodium sulfide (Na_x_S) to prevent its dissolution into electrolytes and the loss of active materials [20]. Figure 8d illustrates the C-rate capability of each sample, the change of specific capacity as a function of the C-rate. Noticeably, the Cu_1.8_S-C/C sample exhibited average specific capacities of 395, 372, 358, 333, 300, and 286 mAh/g at the rates of 0.1, 0.5, 1, 2, 4, and 5 C, respectively, and high cycling stability; these values were superior to the previous reports [3,18,19]. When the C-rate went back to 1 C, the Cu_1.8_S-C/C anode returned back to an average capacity of 345 mAh/g, close to the original value at the same C-rate, suggesting the excellent C-rate capability. However, for the pure Cu_1.8_S-C sample, we found the higher capacities at lower C-rates (0.1, 0.5, and 1 C), but the lower capacities at higher C-rates (2, 4, and 5 C) relative to the Cu_1.8_S-C/C sample. Furthermore, we observed the capacity decay of the Cu_1.8_S-C/C sample with the cycle irrespective of C-rates. The better cycling stability of the Cu_1.8_S-C/C also supports the aforementioned roles of the nanocarbon layers coated on the Cu_1.8_S-C structure.

## 4. Conclusions

We have designed and fabricated a nanoporous Cu_1.8_S-C/C core/shell anode structure with a high surface area by utilizing MOF-199 as a sacrificial template through a two-step process of sulfurization and carbonization via H_2_S gas-assisted PECVD and subsequent hydrothermal-annealing processes. With this anode structure, we achieved a superior SIB performance of ~93% capacity retention in conjunction with a specific capacity of 372 mAh/g after 110 cycles, compared to the pure Cu_1.8_S-C. The higher SIB performance emerged from the decisive contribution of the low-degree graphitic carbon layers covered on the Cu_1.8_S-C to buffer effectively against mechanical stresses induced by volume variation of the Cu_1.8_S-C during cycling, improve electrical conductivity, and tether the Na_x_S to circumvent its dissolution into an electrolyte. The experimental considerations that have hindered this form of battery technology can be resolved with advanced materials. Considering such physico-chemical and electrochemical properties, we confirmed that the Cu_x_S-C/C core/shell structure should be implemented with the ever-increasing development of large-scale and advanced SIBs.

## Supplementary Materials

The following are available online at https://www.mdpi.com/1996-1944/12/8/1324/s1, Figure S1: A dicopper(II) tetracarboxylate building block for MOF-199 with key distances of Cu–Cu 2.628(2) Å, Cu–OCO 1.952(3) Å and Cu–OH_2_ 2.165(8) Å. Figure S2: EDS mapping and spectral analysis for the CuS-C structure sulfurized and carbonized at 350 °C. Notice that the Si and O peaks are associated with the Si/SiO_x_ substrate for the EDS analysis. Figure S3: EDS mapping and spectral analysis for the Cu_1.8_S-C structure sulfurized and carbonized at 550 °C. Figure S4: EDS mapping and spectral analysis for the Cu_2_S-C structure sulfurized and carbonized at 650 °C. Figure S5. EDS mapping and spectral analysis for the as-synthesized MOF-199 structure is shown above. The results are summarized in the below graph in which the bound states of Cu exhibit a higher emission spectra (keV). No extraneous peaks were observed suggesting clean synthesis, processing and purification except the Si peak corresponding to the Si/SiO_x_ substrate. Figure S6: HRTEM images to demonstrate the core/shell structure of the Cu_1.8_S-C/C sample showing the interlayer distance (~0.27 nm) of Cu_1.8_S (200) plane and the thickness (~2.6 nm) of carbon coating layer (C). Figure S7: EDS line scanning results of a Cu_1.8_S-C/C core-shell structure to identify the distribution of elemental S and C present in the structure. Figure S8: Raman spectra of the Cu_1.8_S-C structure sulfurized and carbonized at 550 °C. Figure S9: Cycling performance and its corresponding Coulombic efficiency of the Cu_1.8_S-C/C anode structure at 2 C for 110 cycles.
