# Supplementary material for: Highly Efficient Nanocarbon Coating Layer on the Nanostructured Copper Sulfide-Metal Organic Framework Derived Carbon for Advanced Sodium-Ion Battery Anode
Source: Materials (Basel). 2019 Apr 23;12(8):1324. doi: 10.3390/ma12081324 (PMC6515688; doi:10.3390/ma12081324)
Supplement: Supplementary file 1 [file materials-12-01324-s001.pdf]

materials

Supplementary Materials

Highly Efficient Nanocarbon Coating Layer on the Nanostructured Copper Sulfide-Metal Organic Framework Derived Carbon for Advanced Sodium-Ion Battery Anode

Chiwon Kang [1,*], Yongwoo Lee [2], Ilhwan Kim [3], Seungmin Hyun [3], Tae Hoon Lee [4,5], Soyeong Yun [5], Won-Sub Yoon [5], Youngkwang Moon [6], Jinkee Lee [6], Sunkook Kim [1] and Hoo-Jeong Lee [1,*]

1 School of Advanced Materials Science and Engineering, Sungkyunkwan University (SKKU), Suwon 16419, Korea; seonkuk@skku.edu
2 Department of Chemistry, University of Massachusetts Lowell, One University Avenue, Lowell, MA, 01854, USA; Yongwoo_Lee@uml.edu
3 Department of Applied Nano Mechanics, Korea Institute of Machinery and Materials (KIMM), Daejeon 305-343, Korea; kihwan20@kimm.re.kr (I.K.); hyun@kimm.re.kr (S.H.)
4 Center for Integrated Nanostructure Physics (CINAP), Institute for Basic Science (IBS), Suwon 16419, Korea; hooni0629@skku.edu
5 Department of Energy Science, Sungkyunkwan University (SKKU), Suwon 16419, Korea; so116102@gmail.com (S.Y.); wsyoon@skku.edu (W.-S.Y.)
6 School of Mechanical Engineering, Sungkyunkwan University, Suwon 16419, Korea; fire6568@skku.edu (Y.M.); lee.jinkee@skku.edu (J.L.);
* Correspondence: chiwonkang@skku.edu (C.K.); hlee@skku.edu (H.-J.L.); Tel.: +82-31-299-4735 (C.K.); +82-31-290-7365 (H.-J.L.)

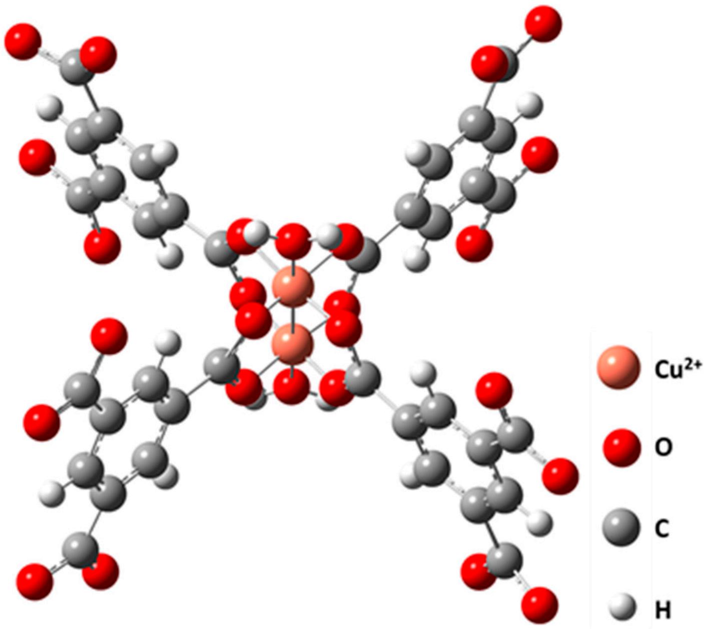

Figure S1. A dicopper(II) tetracarboxylate building block for MOF-199 with key distances of Cu–Cu 2.628(2) Å, Cu–OCO 1.952(3) Å and Cu–OH_2 2.165(8) Å.

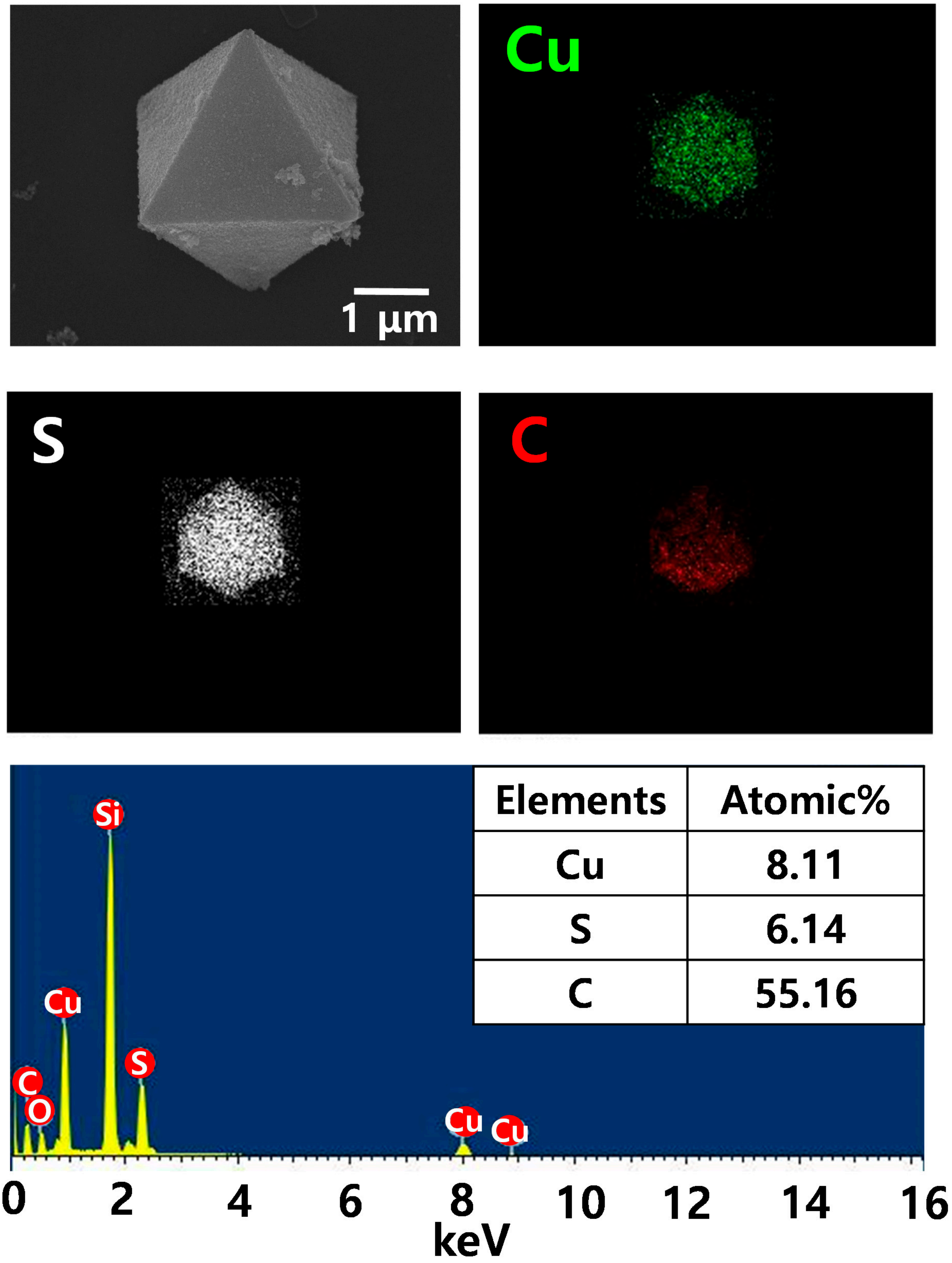

Figure S2. EDS mapping and spectral analysis for the CuS-C structure sulfurized and carbonized at 350 °C. Notice that the Si and O peaks are associated with the Si/SiO_x substrate for the EDS analysis.

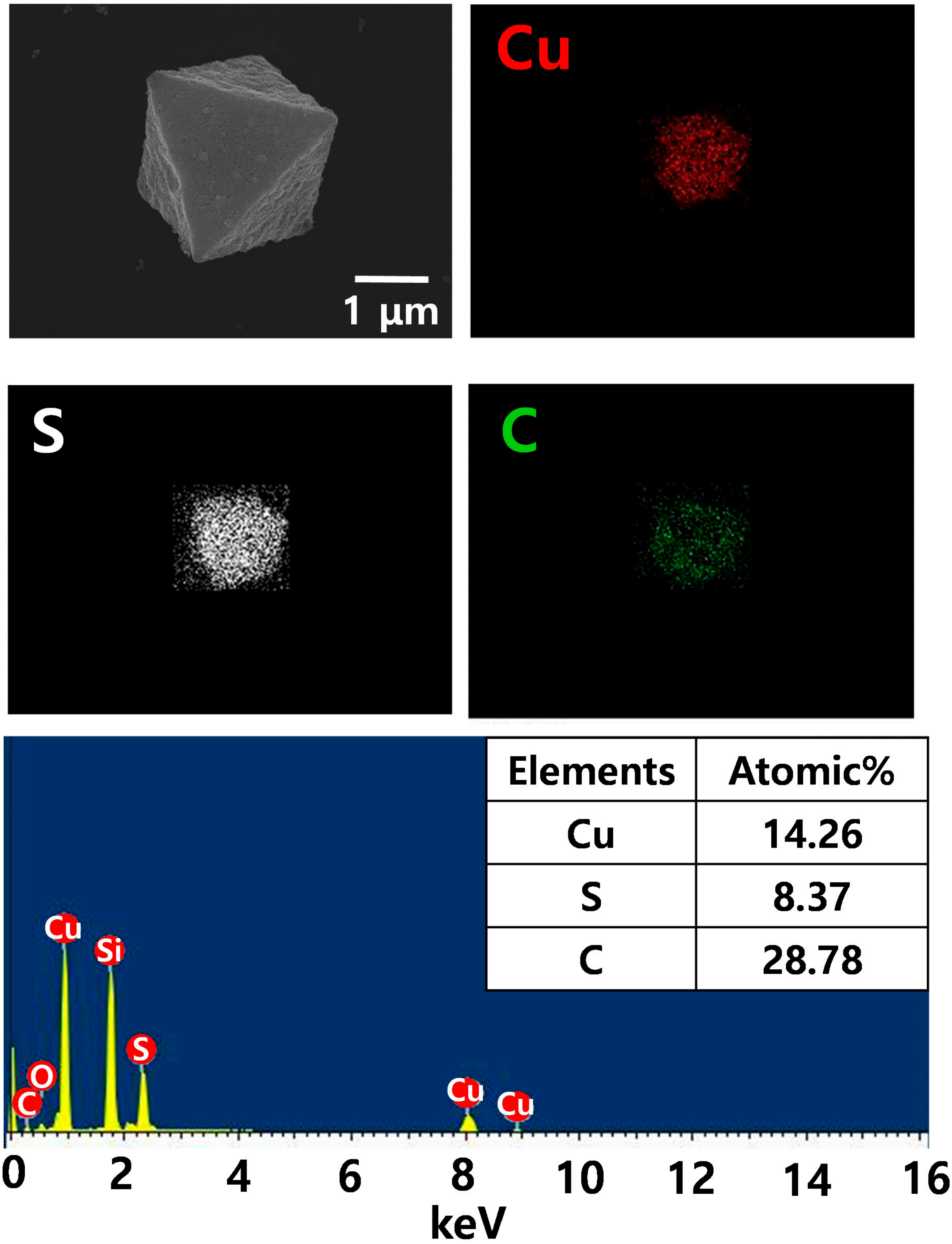

Figure S3. EDS mapping and spectral analysis for the $Cu_{1.8}S$-C structure sulfurized and carbonized at 550 °C.

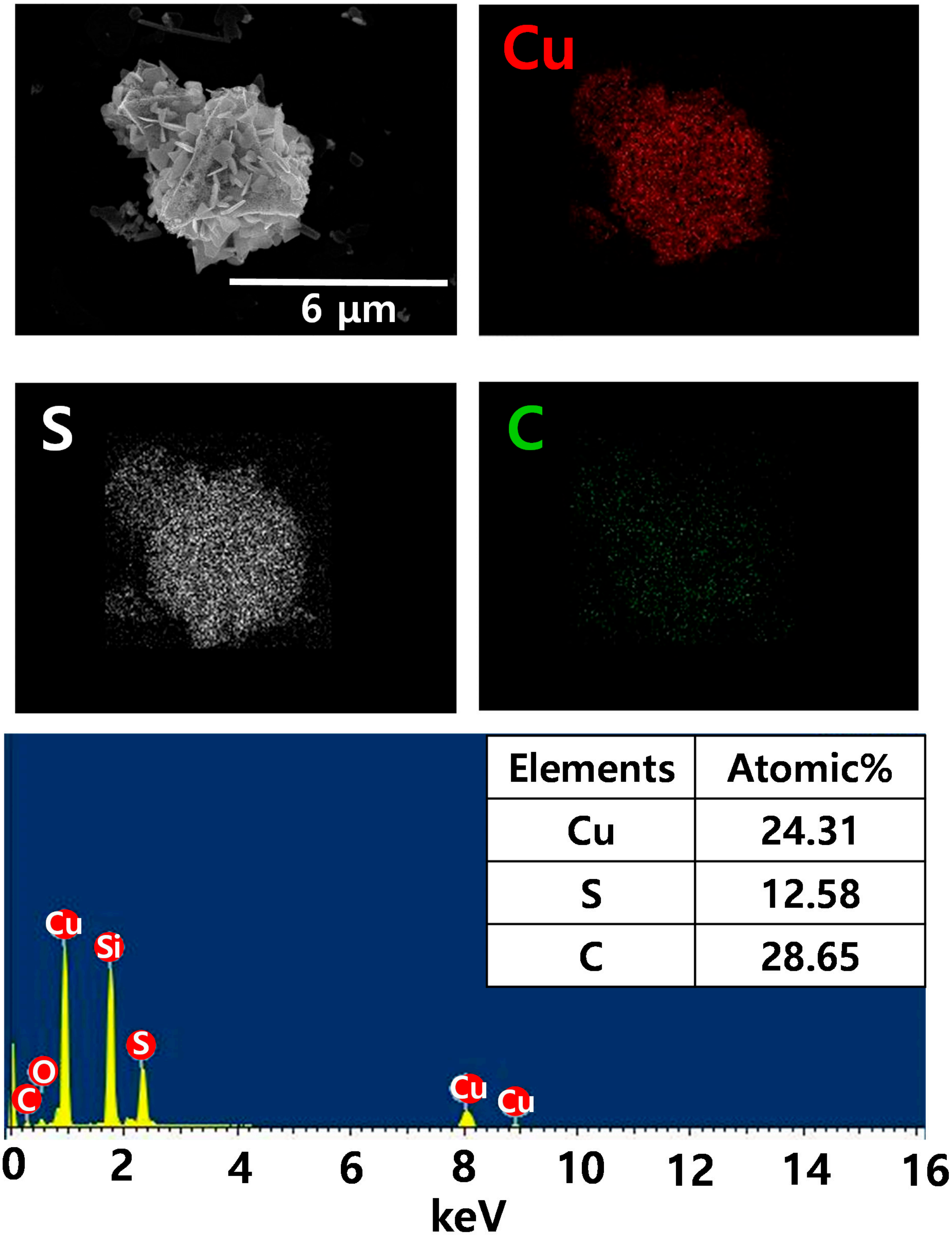

Figure S4. EDS mapping and spectral analysis for the Cu_2S-C structure sulfurized and carbonized at 650 °C.

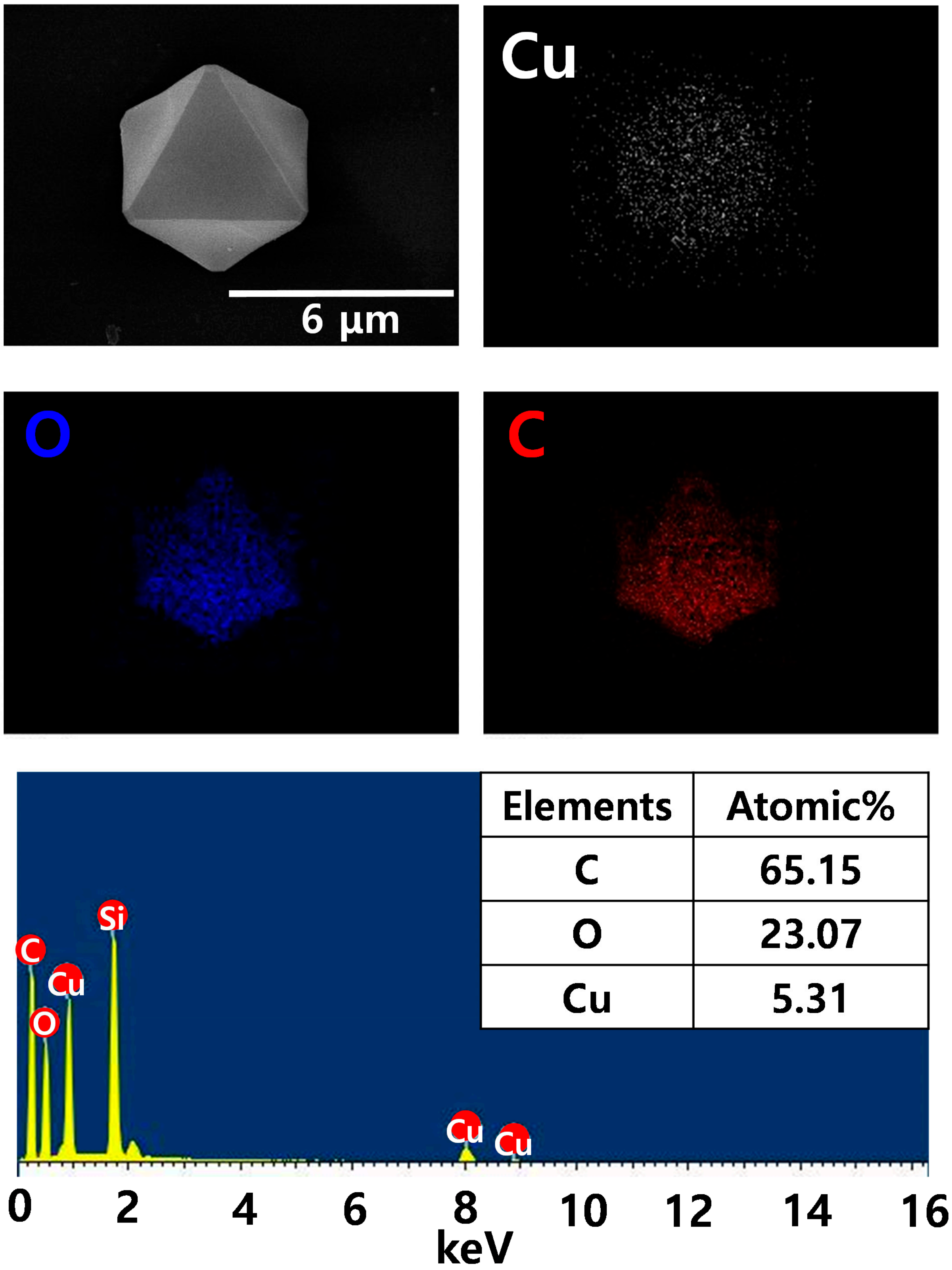

Figure S5. EDS mapping and spectral analysis for the as-synthesized MOF-199 structure is shown above. The results are summarized in the below graph in which the bound states of Cu exhibit a higher emission spectra (keV). No extraneous peaks were observed suggesting clean synthesis, processing and purification except the Si peak corresponding to the Si/SiO_x substrate.

The mechanism of multistep reaction of Cu_xS-C with oxygen

For the CuS-C sample, the mass drop occurred at the temperature ranging from 150 to 222 °C, which is associated with the oxidation of amorphous carbon and the emission of SO_2 gas as by-products during the conversion of CuS to Cu_2S (Eq. 1).

$$2CuS + O_2 \rightarrow Cu_2S + SO_2 \qquad \text{...... (Eq. 1)}$$

The Cu_9S_5 ($Cu_{1.8}S$) in the Cu_9S_5-C sample was transformed to Cu_2S (Eq. 2).

$$2Cu_9S_5 + O_2 \rightarrow 9Cu_2S + SO_2 \qquad \text{...... (Eq. 2)}$$

After both samples (CuS-C and $Cu_{1.8}S$-C) were converted to Cu_2S-C, they thermally behaved like the Cu_2S-C sample, in which Cu_2S was oxidized to Cu_2O (Eq. 3).

$$2Cu_2S + 3O_2 \rightarrow 2Cu_2O + 2SO_2 \qquad \text{...... (Eq. 3)}$$

We observed a slight mass drop (~9 wt%) present in the TGA curve for the Cu_2S-C sample; this is attributed to the oxidation of the carbon in the temperature range of 296 to 337 °C.

From 210 °C onwards, all the three samples showed a mass uptake, proportional to the amount of Cu_xS content present in each sample. This response is due to the conversion of Cu_2S to intermediate copper sulphate ($CuO{\cdot}CuSO_4$) as expressed in the Eq. 4.

$$Cu_2S + SO_2 + O_2 \rightarrow CuO \cdot CuSO_4 \qquad \text{...... (Eq. 4)}$$

For the Cu-rich ($Cu_{1.8}S$-C and Cu_2S-C) and Cu-poor phase (CuS-C) samples, the copper sulphates were stable up to ~640 and ~600 °C, respectively. Afterwards, the mass drops of CuS-C, $Cu_{1.8}S$-C and Cu_2S-C samples occurred until temperatures rose to 742, 820, and 778 °C, respectively (Eq. 5 and 6).

$$2CuO \cdot CuSO_4 \rightarrow 4CuO + 2SO_2 + O_2 \qquad \text{...... (Eq. 5)}$$

$$4CuO \rightarrow 2Cu_2O + O_2 \qquad \text{...... (Eq. 6)}$$

Finally, we observed no significant weight loss of all the samples up to 900 °C, resulting in the weight losses of ~68% (CuS-C), ~69% ($Cu_{1.8}S$-C) and ~89% (Cu_2S-C), respectively (see Figure 4 in the manuscript). Based on this mechanism, we calculated the mass fraction of Cu_xS in Cu_xS-C: ~46% (CuS-C), ~77% ($Cu_{1.8}S$-C) and ~100% (Cu_2S-C), respectively, according to the residual molecular weight of 89% for the conversion of Cu_2S to Cu_2O and 148% for the conversion of CuS to Cu_2O. Despite the calculated mass fraction (~100%) for the Cu_2S-C sample, we confirmed the

presence of carbon in the sample by the EDS mapping results (Figure S4). Therefore, we determined a mass fraction of ~91% for the Cu_2S in the Cu_2S-C sample based on the aforementioned mass drop (~9 wt%) of the TGA curve of the Cu_2S-C sample.

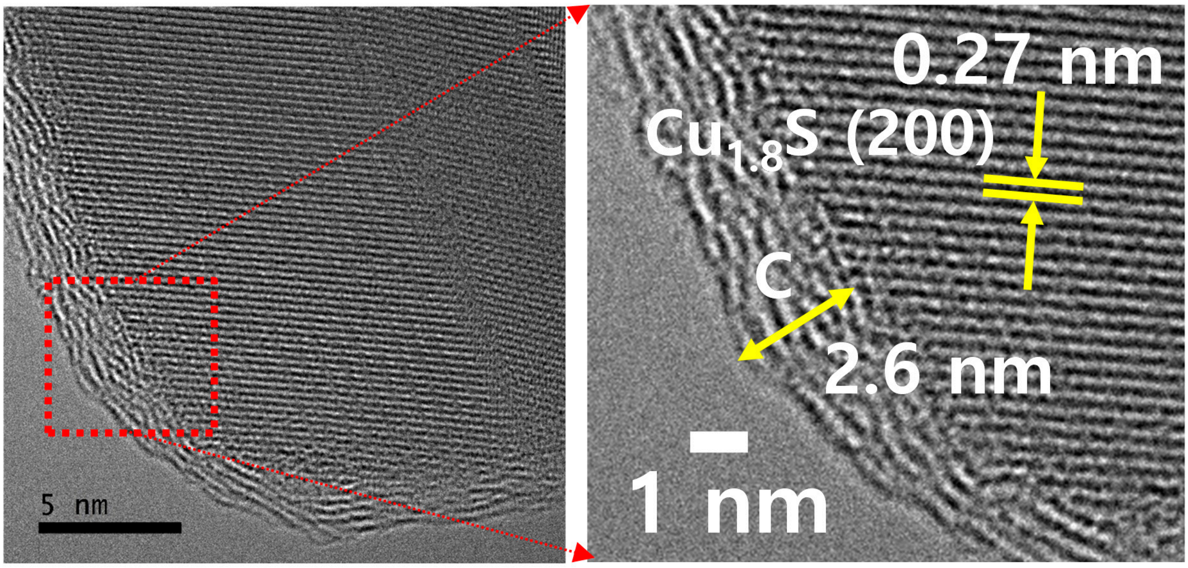

Figure S6. HRTEM images to demonstrate the core/shell structure of the $Cu_{1.8}S$-C/C sample showing the interlayer distance (~0.27 nm) of $Cu_{1.8}S$ (200) plane and the thickness (~2.6 nm) of carbon coating layer (C).

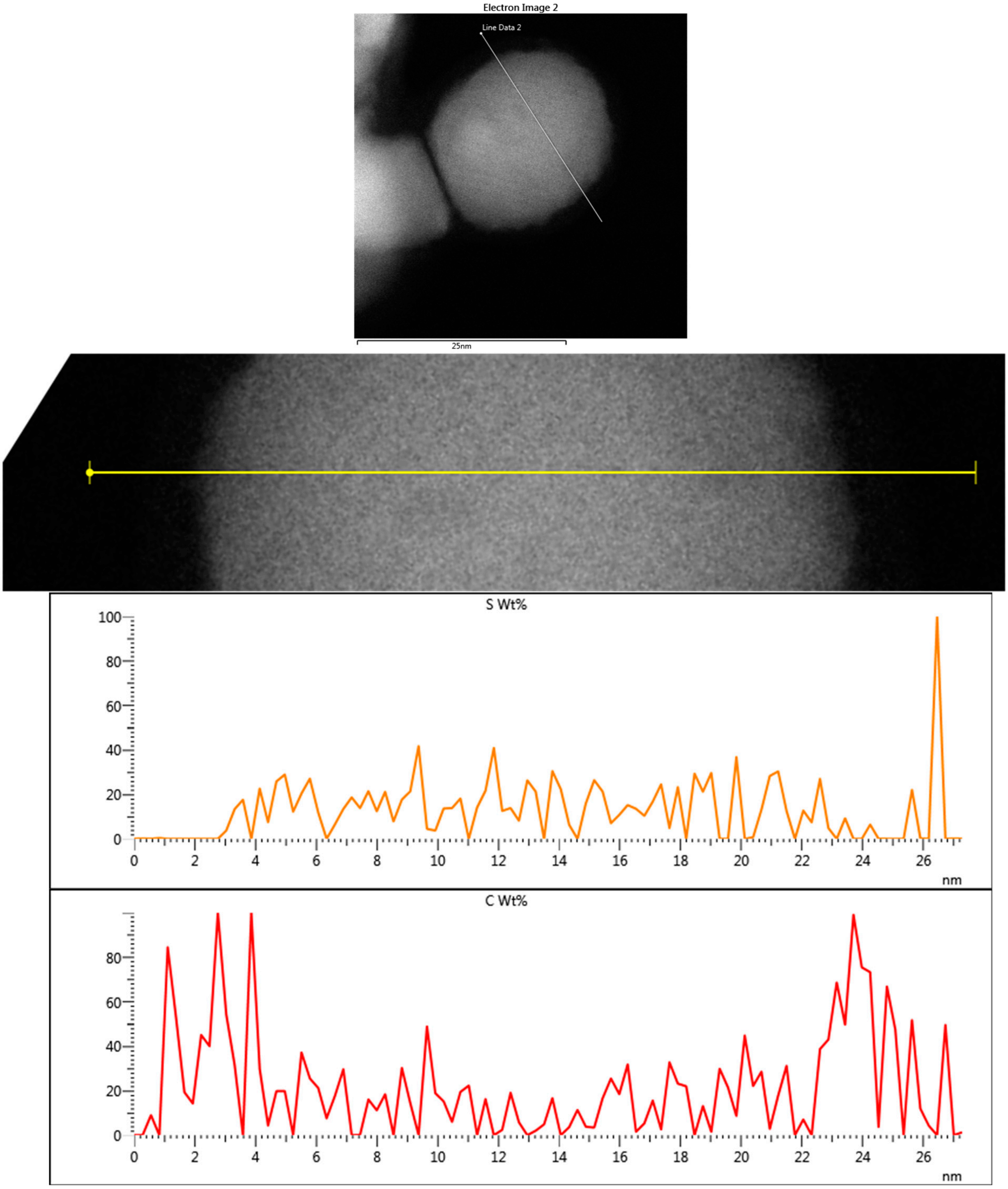

Figure S7. EDS line scanning results of a $Cu_{1.8}S$-C/C core-shell structure to identify the distribution of elemental S and C present in the structure.

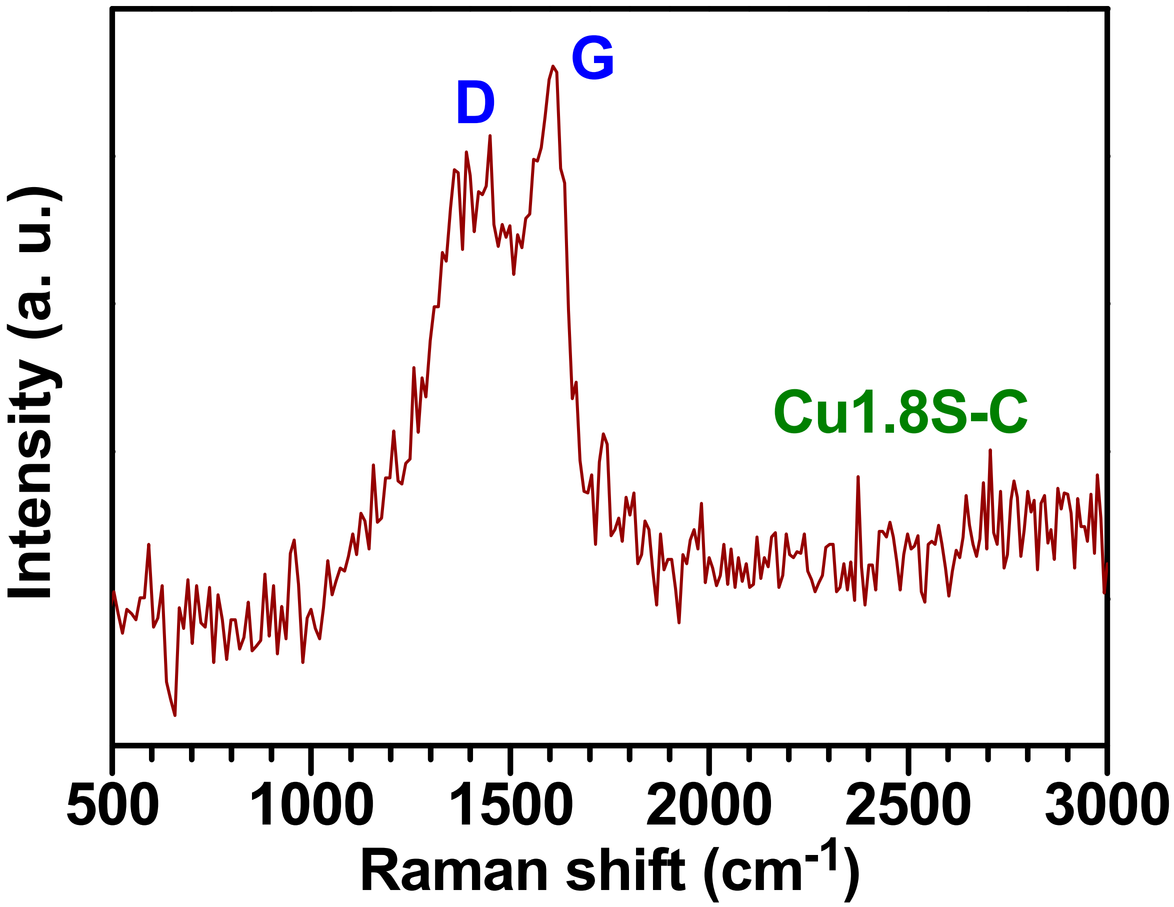

Figure S8. Raman spectra of the $Cu_{1.8}S$-C structure sulfurized and carbonized at 550 °C.

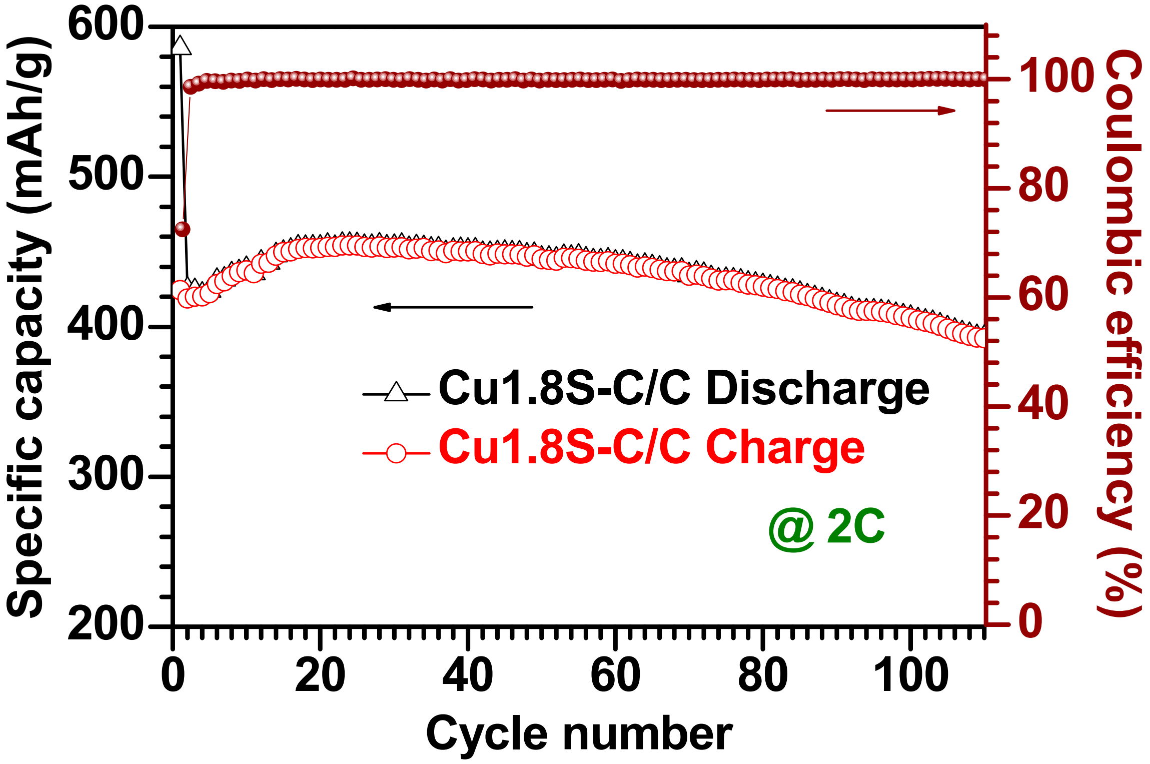

Figure S9. Cycling performance and its corresponding Coulombic efficiency of the $Cu_{1.8}S$-C/C anode structure at 2C for 110 cycles.

© 2019 by the authors. Submitted for possible open access publication under the terms and conditions of the Creative Commons Attribution (CC BY) license (http://creativecommons.org/licenses/by/4.0/).
